# Seeds from ALS patients homozygous for the SOD1 D90A mutation transmit two types of SOD1 aggregation and motor neuron disease

**DOI:** 10.1007/s00401-026-03078-3

**Published:** 2026-09-01

**Authors:** Caitlin Henne, Isabelle Sigfridsson, Thomas Brännström, Karin M. E. Forsberg, Stefan L. Marklund, Per Zetterström, Peter M. Andersen

**Affiliations:** 1 Department of Medical Biosciences, Umeå University, 901 87 Umeå, Sweden; 2 Department of Clinical Sciences, Neuroscience, Umeå University, 901 87 Umeå, Sweden

**Keywords:** ALS, SOD1, D90A, Prion, Transmission

## Abstract

Mutations in superoxide dismutase−1 (SOD1) are a common cause of amyotrophic lateral sclerosis (ALS). Inheritance is as a rule dominant, but in carriers of the most prevalent mutation, D90A, disease primarily develops in homozygotes. Increasing evidence suggests that prion-like propagation of SOD1 aggregation is the central pathogenic mechanism. Two structurally different strains of aggregates have been found to arise in human SOD1 (hSOD1) transgenic (Tg) mouse models of ALS. Strain A is formed by most mutants including hSOD1^G85R^ and homozygous hSOD1^WT^ Tg mice, whereas homozygous hSOD1^D90A^ Tg mice form a distinct strain B, but also A. Inoculation of strain A and B seed preparations from Tg mice into lumbar spinal cord of adult hSOD1^G85R^ mice induced templated spreading hSOD1 aggregation and premature ALS-like disease. Seeds from an ALS patient carrying the hSOD1^G127X^ truncation mutation likewise transmitted strain A aggregation and disease. In the present study, we investigated whether seeds prepared from spinal ventral horns from six patients homozygous for the hSOD1^D90A^ mutation could transmit aggregation and disease to adult hSOD1^G85R^ Tg mice. Despite the extensive degeneration and loss of motor neurons in the long-lived D90A patients, two of the seeds significantly shortened the survival of the Tg mice, one transmitting A and the other B-pattern hSOD1 aggregation. Nine different preparations from four human controls lacked effects. The results demonstrate that two distinct aggregate strains can arise and propagate in homozygous hSOD1^D90A^ ALS patients, further supporting the hypothesis that prion-like transmission of hSOD1 aggregation is the primary pathogenic mechanism in SOD1-linked ALS.

**Supplementary Information:**

The online version contains supplementary material available at https://doi.org/10.1007/s00401-026-03078-3.

## Introduction

Amyotrophic lateral sclerosis (ALS) is characterized by adult-onset degeneration of upper and lower motor neurons. The disease begins focally and then spreads contiguously, resulting in progressive paralysis and death from respiratory failure [11]. Mutations in the gene encoding the ubiquitously expressed free radical scavenging enzyme superoxide dismutase−1 (SOD1) can cause ALS [27] and are found in 1–9% of patients [1]. Since 1993, over 235 coding mutations in *SOD1* have been associated with ALS as a dominant trait (http://alsod.iop.kcl.ac.uk/) [29]. However, disease caused by the most prevalent SOD1 mutation, D90A, is most frequently inherited as a recessive trait with a uniform slowly ascending phenotype [2, 3], but may rarely be inherited as a dominant trait with a variable phenotype for first symptom and survival time [2, 3, 26].

Cytosolic inclusions containing aggregated SOD1 are hallmarks of ALS both in patients and transgenic (Tg) animal models expressing mutant human SOD1s (hSOD1) [20]. Using a binary epitope mapping method (BEM), we have found that two different strains of hSOD1 aggregates, A and B, can arise in mice that express hSOD1 variants [8]. The hSOD1 variants were found to have different propensities in forming the aggregate strains. Strain A aggregates appeared in mice that hemizygously express G93A mutant hSOD1 (hSOD1^G93A^), hSOD1^G85R^ and homozygously express hSOD1^WT^. In contrast homozygous hSOD1^D90A^ mice form a distinct strain B, but also A. Inoculation of strain A or B aggregate seed preparations into spinal cords of mice expressing a hSOD1 transgene induced templated spreading hSOD1 aggregation. The process was accompanied by premature fatal motor neuron disease with hallmarks closely resembling human ALS [9]. Seeding effects of spinal cord homogenates from end-stage Tg mice have also been demonstrated in Tg mice as well as in cultured cells and in vitro [5–7, 12, 21, 22, 25]. Moreover, preparations from spinal cords of ALS patients with the aggressive *SOD1* G127X, A4V and N139K mutations have also been found to seed Tg mice [13, 30]. The seed from the SOD1^G127X^ patient transmitted strain A aggregation. These findings suggest that prion-like spreading of hSOD1 aggregation could be the primary pathogenic mechanism of hSOD1-induced ALS. 

Here we examined for the first time whether seeds from ALS patients homozygous for the hSOD1^D90A^ mutation also could transmit the disease to hSOD1 Tg mice and, if so, which strain patterns would be templated. A patient with the hSOD1^G127X^ mutation was chosen in our previous study because carriers of this mutation have short survivals and the spinal ventral horns have been found to contain relatively large amounts of mutant aggregates in remaining motor neurons [17, 18]. In contrast, patients homozygous for the hSOD1^D90A^ mutation may survive into decades (median 14 years) [3] and post-mortem examination show the ventral horns in the entire spinal cord to be severely degenerated with profound motor neuron loss [16]. Thus, seeds prepared from the ventral horns are likely to contain only minimal amounts of remaining motor area cell material. We inoculated such seeds from six patients as well as nine different preparations from four controls into lumbar spinal cord of adult hSOD1^G85R^ Tg mice. Two of the hSOD1^D90A^ patient-derived seeds shortened the survival of the Tg mice significantly, one transmitting A and the other B-pattern aggregation in some of the mice. In contrast Tg mice inoculated with the nine control preparations showed lifespans indistinguishable from non-inoculated mice.

## Materials and methods

### Mice

In this study we used hSOD1^G85R^ Tg mice hemizygous for the insertion [10]. The strain was backcrossed >30 generations in C57BL/6 mice. The use and maintenance of the mice and the experimental protocol described in this article were approved by the Umeå Regional Ethics Committee for Animal Research (A28-2018).

### Human material and preparation of seeds

Clinical and histopathological details of the 6 patients homozygous for the hSOD1^D90A^ mutation are presented as patients # 1, 5, 3, 7, 2 and 4 in [16] and are summarized here in Table 1 as patients A1 through A6. The 4 control individuals are listed in Table 2. The postmortem time was significantly shorter for the ALS patients (*p*=0.019), but the death age was not (*p*=0.352). All autopsies were performed by the same staff at Umeå University Hospital. From all 10 individuals, seeds were prepared from lumbar ventral horn specimens, including the whole lamina IX. Three different protocols for seed preparation were used. The six hSOD1^D90A^ samples were prepared according to protocol I. Seeds from one control were prepared using all three protocols (C1). Seeds from the remaining three controls were prepared using both protocols II and III.

**Table 1:** Characteristics of D90A-ALS patients and seeds

Patient characteristics						Seed characteristics		
Patient	Sex	Death age (years)	Disease duration (months)	Postmortem time (hours)	Autopsy year	Seed protocol	SOD1 (µg/ml)	Total protein (µg/ml)
A1	F	43.3	60	29	2000	I	0.069	1936
A2	M	70.3	176	48	2008	I	0.032	4089
A3	F	63.9	131	72	2005	I	0.055	2676
A4	M	74.8	316	48	2005	I	0.011	2235
A5	M	66.2	102	48	2003	I	0.061	3472
A6	M	53.5	265	48	2001	I	0.023	2513

**Table 2:** Characteristics of control patients and seeds

		Patient characteristics					Seed characteristics		
Patient	Sex		Death age (years)	Postmortem time (hours)	Autopsy year	Cause of death	Seed protocol	SOD1 (µg/ml)	Total protein (µg/ml)
C1	M		73.0	30	2008	Post-stroke epilepsy	I	0.050	2676
							II	0.010	8677
							III	7.70	1844
C2	M		63.6	<30	2005	Multiple sclerosis	II	0.014	19,425
							III	12.2	3350
C3	F		80.8	18	2011	Atypical parkinsonism	II	0.012	11,620
							III	12.0	3251
C4	M		83.7	28	2018	Myocardial infarction	II	0.015	5023
							III	10.1	3439

I. Briefly, the protocol involved homogenization in 12.5 volumes of PBS containing 1% NP-40, 1 M guanidinium chloride using an Ultraturrax and sonication [9]. After clearing by 1000 × g centrifugation, the homogenates were layered onto 4 cm high 13% Iohexol cushions (*δ*=1.074) and centrifuged at 175,000xg for 60 min. Under these conditions, proteinaceous components with molecular masses >~5 × 10^6^ Da will be pelleted [28]. The pellets were suspended by sonication in a small volume of PBS (~ 75% volume, µl PBS/mg tissue) and stored at −80 ºC.

II. Similar to I, but 0.5 M guanidinium chloride was used instead of 1 M. Following sonication, the homogenate was diluted with 3.33 vol water with 1% NP40. After sonication, the homogenates were loaded onto 4 cm 13% iohexol cushions and further processed as in protocol I.

III. Specimens from the four controls were also homogenized in 7 volumes (w/v) of PBS using an Ultraturrax (IKA) followed by sonication for 1 min and centrifugation at 1000 × g at 4 ºC for 10 min. The resulting supernatants were stored at −80 ºC.

### Inoculation of the ventral horn seeds into lumbar spinal cord and monitoring of mice

The protocol used was somewhat modified compared with the previous study [9]. The recipient 100-day-old asymptomatic *hSOD1*^*G85R*^ Tg mice were anaesthetized with 5% isoflurane in an induction chamber. The mice were then fixed on a small animal stereotactic frame (Kopf Instruments, Tuiunga. Ca, USA) and anaesthesia maintained with 1.5–2% isoflurane via a facemask. Also, 0.005 µg Rimadyl per gram weight was injected s.c. approximately 15 min before starting surgery. Two small bilateral, longitudinal cuts on the back close to the spine were then made, making pockets for attachment of a stabilizing spine clamp. To reach the spinal cord, a 3–4 mm transversal cut through the muscles was made. Inoculation was done between two vertebrae after the meninges were punctured using a fine needle. The depth of insertion was controlled using a digital display console with 10 µm resolution (model 940-B, Kopf). One µl of the seeds were inoculated into the lumbar ventral horn on the left side at the L2-L3 levels. A Syringe model 75 RN Neuros with a sharp custom syringe (33/78/4) S 30˚ (Hamilton, Bonaduz, Switzerland) was used, and the injection velocity was 0.125 µl/min controlled by an infusion pump (Legato 130, KD Scientific). The syringe was then slowly pulled out, the fascia sutured, and the skin closed using Tissue Adhesive (3 M Vetbond, Haliston, MA, USA). The duration of the surgical procedures was about 25 min, and in total the mice were under anaesthesia for about 40 min. To compensate for dehydration and any blood loss, 250 µl physiological NaCl was injected subcutaneously. Buprenorphine was injected 2–3 times at 12-h intervals until the mice were pain free.

No obvious sphincter or other bladder disturbances were observed in any of the operated mice, and the sensory response in their hind legs was normal. The mice were examined at least every third day and weighed once a week. The mice were considered terminally ill when the hind legs were fully or almost fully paralyzed. In some of the mice, the paralysis was more prominent in the forelimbs. In these cases, the end stage was set to when the paralysis was so extensive that the mice could not eat wet food or developed an eye infection.

### Tissue collection for BEM and immunohistochemistry

Mice were sacrificed following a lethal i.p. injection of pentobarbital (60 mg/mL, APL Pharma, Stockholm, Sweden) and were perfused transcardially with PBS. The brain including the medulla was dissected from the skull, while the spinal cord was quickly retrieved by flushing the vertebral canal with ice-cold saline using a syringe inserted caudally to sacral levels. The medulla was then separated from the brainstem and divided sagittally into right and left sides. The spinal cord was divided into lumbar, thoracic, cervical segments before undergoing the same sagittal division. Tissues from one sagittal half, alternating ipsi-, and contralateral sides, were snap-frozen in liquid nitrogen and stored at − 80° C for biochemical analyses and BEM. Tissues from the other half were fixed in 10% buffered formalin, dehydrated and paraffin-embedded for immunohistochemistry.

For BEM analysis, the tissue segments were homogenized with an Ultraturrax apparatus (IKA, Staufen, Germany) for 30 s followed by sonication for 1 min in 25 volumes of ice-cold PBS containing an antiproteolytic cocktail (Complete, Roche Diagnostics, Basel, Switzerland). The tissue homogenates were then added to 20 volumes of PBS containing 1% polyethylene glycol nonylphenyl ether (NP40), 1 mM dithiothreitol (DTT), 1.8 mM EDTA and Complete EDTA-free, sonicated for 30 s, and cleared by centrifugation at 1000 × g for 10 min.

### Identities of analyzed mice

In some cases, the post-inoculation lifespan order of analyzed mice is of importance for interpretation and will be presented. E.g. A1:1 means the first mouse to become terminally ill of those inoculated with the A1 seed.

### Antibodies

Antibodies to peptides in hSOD1 (corresponding to amino acids (aa) 4–20, 24–39, 43–57, 57–72, 80–96, 100–115, and 131–153) that were coupled to keyhole limpet hemocyanin were raised in rabbits and affinity purified with immobilized peptides as described [14 Jonsson, 2004 #13]. An antibody raised to the C-terminal end in hSOD1^G127X^ including the non-native sequence (aa 111–127GQRWK) was prepared in the same way. It will only react with aas 111–127 in the full-length hSOD1s and is presented so in the figures.

### Binary epitope-mapping assay for hSOD1 aggregate structure

A detailed description of the protocol and background to the BEM method is found in [8]. The protocol is based on eight polyclonal rabbit antibodies raised against short peptides covering the 153 amino acids long hSOD1 subunits. The reaction of the antibodies with aggregates captured on the filter is determined. Since the configurational space of short peptides is very large, their randomly induced antibody-eliciting epitopes are unlikely to match defined ordered structures in proteins and in cores of protein fibrils. The antibodies have been found to lack reactivity with natively folded SOD1. In contrast, all react avidly with denatured/disordered SOD1, in which the corresponding peptide segments can adapt to the antigen-binding sites [14, 15]. In reaction with fibrils/aggregates, the binding of the anti-peptide antibodies is essentially “binary”. There is no response to the ordered core of protein aggregates/fibrils or to segments otherwise hidden. Non-recruited sequence elements, which have lost their native contacts and, therefore, are disordered, will react with the antibodies.

Aggregates in serial 1 + 1 dilutions of the preparations to be analyzed were trapped in 0.2 µm cellulose acetate filters in a dot-blot apparatus. All samples were run in duplicates. The filters were washed three times with PBS. They were then cut into slices, incubated with the 8 antibodies overnight and finally developed, such aswestern immunoblots. Chemiluminescence of the blots was recorded in a ChemiDoc Touch Imaging System (BioRad, Hercules, CA, USA) and analyzed with ImageLab software. To allow comparison and quantification, one homogenate of spinal cord from an end-stage *hSOD1*^*G93A*^ Tg mouse, kept frozen in aliquots, is designated as a standard (set to 1) and run in one or two lanes on all filters and stained with the aa 57–72 antibody. All blots of all preparations with all antibodies were quantified against this standard. Examples of blots showing strain A and strain B patterns are presented in Supplementary Fig. S1. To facilitate comparison of BEM staining patterns, the staining intensities of the eight antibodies with individual preparations were normalized against the staining of that preparation with the aa 57–72 antibody (taken as 100%).

### Quantification of SOD1

The amount of SOD1 in the different seeds was quantified using quantitative immunoblotting as described before [23]. As SOD1 standard, a human hemolysate was used, with the SOD1 content calibrated against pure human SOD1, the concentration of which was determined by quantitative amino acid analysis [24]. Shortly, the seeds and standards were diluted in sample buffer for SDS-PAGE containing β-mercaptoethanol and separated by SDS-PAGE using Criterion TGX Stain-Free Any-KD precast gels (Bio-Rad Laboratories), transferred to 0.2 µm nitrocellulose membranes using Trans-Blot Turbo Midi Nitrocellulose transfer packs (Bio-Rad) using a Trans-Blot Turbo Transfer System (Bio-Rad). The aa 24–39 antibody was used as primary antibody at a concentration of 1 µg/mL, and the secondary antibody was an HRP-conjugated polyclonal goat anti-rabbit antibody (P0440 by DAKO) both diluted in 20 mM TBST containing 0.1% Tween-20 and 5% non-fat dry dissolved milk powder. The chemiluminescent reagent was ECL Select (Cytiva), the ChemiDoc Touch Imager (Bio-Rad) was used, and evaluation and quantification of the band intensities was done with the ImageLab software (Bio-Rad).

### Total protein quantification

The total amount of protein was quantified using the Pierce BCA protein assay kit (ThermoFisher Scientific), following the standard protocol provided by the supplier. Pierce Bovine Serum Albumin Standard Ampules at 2 mg/mL in 0.9% NaCl (ThermoFisher Scientific) stored at −80 °C were used as protein standard.

### Histopathology for strain A and B aggregates

The formalin-fixed and paraffin-embedded specimens were cut to 5 µm thick sections using a microtome and mounted on glass slides (Superfrost^®^, ThermoFisher Scientific). The tissues were immunostained according to the manufacturer’s recommendations using the automated stainer Discovery Ultra (Roche Diagnostics). Single immunostainings targeting aa 57–72 or aa 111–127 were performed, as well as double-immunolabeling using both antibodies to distinguish both strain A and B in the same tissue.

For single immunostainings, the tissues were treated with enzymatic antigen retrieval (Ventana Protease 1) for 4 min before incubation with the aa 57–72 antibody (0.83 µg/ml) or aa 111–127 antibody (0.86 µg/ml). The primary antibody was detected using anti-rabbit secondary antibodies conjugated with horseradish peroxidase (Omni-Map, Roche Diagnostics) and visualized using DAB staining (ChromoMap DAB detection kit, Roche Diagnostics). The sections were counterstained with Hematoxylin II (Roche Diagnostics), washed, dehydrated and finally mounted with Pertex (DakoCytomation). Images were obtained using Olympus BX61 microscope and the Olympus cellSens Standard software version 3.2. 

For the double labeling, the tissues were treated with enzymatic antigen retrieval (Ventana Protease 1) for 4 min before incubation with the primary antibody targeting aa 111–127 (0.86 µg/ml). The primary antibody was detected using anti-rabbit secondary antibodies conjugated with horseradish peroxidase (Omni-Map, Roche Diagnostics) and visualized using Rhodamine kit (Roche Diagnostics). The tissues were incubated at 100 ºC for 8 min to denature the previously added antibodies before adding the primary antibody targeting aa 57–72 (0.83 µg/ml). The aa 57–72 antibody was detected using an anti-rabbit secondary antibody conjugated with horseradish peroxidase (Omni-Map, Roche Diagnostics) and visualized using FITC kit (Roche Diagnostics). The sections were counterstained with DAPI (Roche Diagnostics), washed and mounted with Fluorescence mounting medium (DAKO). Images were obtained using Zeiss 710 confocal laser scanning microscope.

### Excluded mice

Nine operated mice were excluded from this study and did not contribute data to any of the included results or statistical analysis. One of the excluded animals was inoculated with the A1 ALS seed and did not display any typical ALS symptoms, but did develop abnormal respiration, leading to it being harvested 232 days after the surgery. There were negligible amounts of aggregates upon BEM analysis (< 5% of the amounts found in paralytic Tg mice). The remaining eight mice received control patient material. Of these, six suffered from non-surgical wounds and were euthanized at 36, 47, 52, 73, and 188 days post-inoculation. The final two excluded animals were found dead in their cages with no prior symptoms noted at 230 and 295 days post-inoculation, respectively.

### Statistical analysis

Statistical analysis of survival data was performed using JASP (Version 0.95.1). Kaplan–Meier curves were generated using ggsurvfit: R-packet version 1.2.0.9001. Kaplan–Meier survival significance was determined using the log-rank Mantel–Haenszel test. Differences in postmortem time and death age were analyzed using the Independent-Samples Mann–Whitney *U* Test. The significance level was set to 0.05.

## Results

### Seeds from homozygous hSOD1^D90A^ patients transmit motor neuron disease

**Fig. 1:**
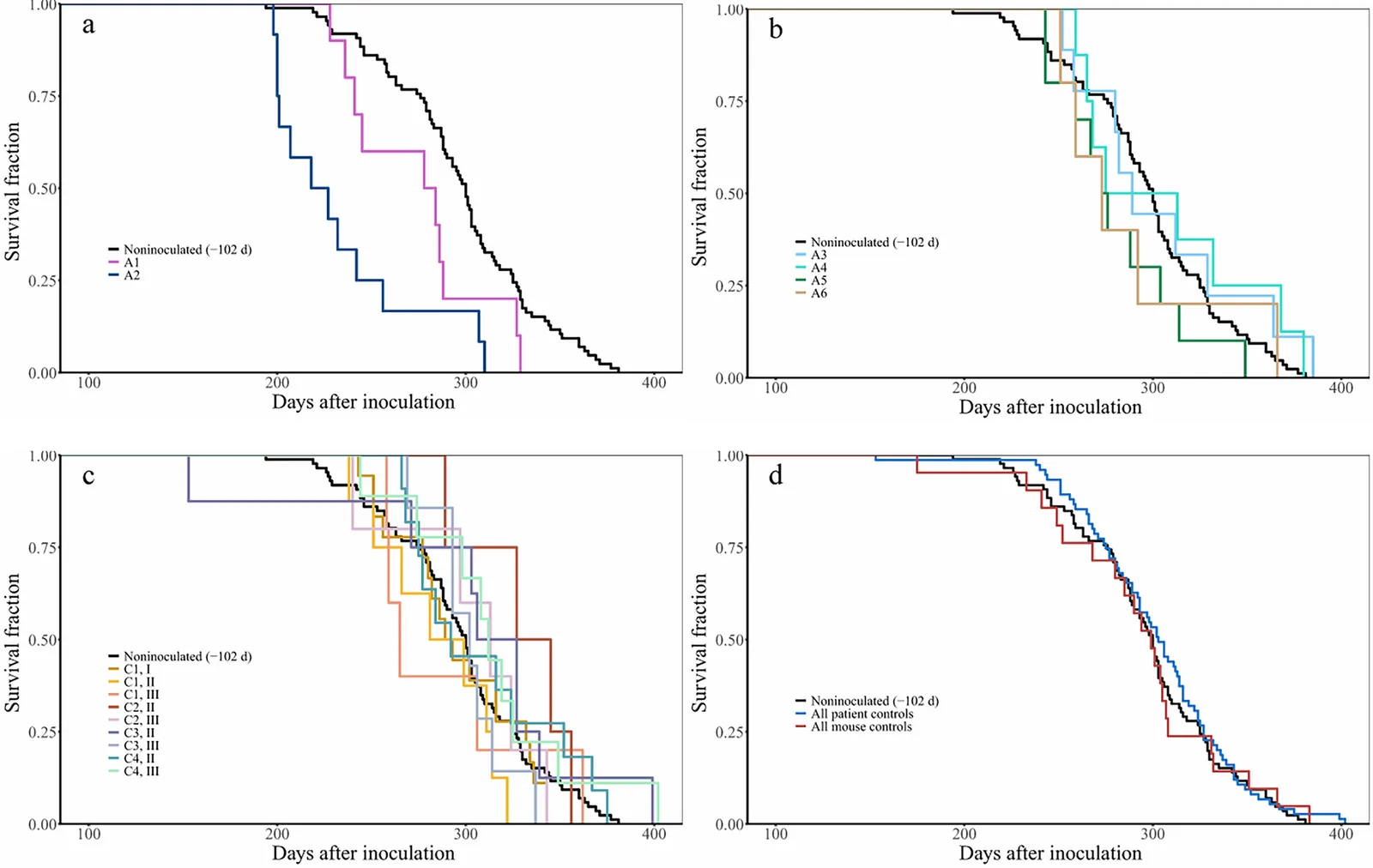
Survival of hSOD1^G85R^ Tg mice inoculated with seeds from D90A patients or control preparations. The survival length of the non-inoculated mice (n=86) was subtracted with 102 d, the mean age at which the mice were inoculated.** a** Two of six seeds from homozygous D90A patients, A1 and A2, shortened the lifespans significantly, whereas, **b**, the remaining four did not. **c** Nine different seeds from four controls lacked significant effects on survival. **d** Pooled data from the 75 mice inoculated with the nine human control seeds show survivals close to non-inoculated controls. Pooled data from 21 mice inoculated with mouse control seeds show likewise absence of effects: 10 prepared according to protocol 1 [9], 7 prepared according to protocol III [21] and 4 prepared with a double gradient protocol (Supplementary Fig. S2)

Previous studies from our group demonstrated that spinal inoculation of hSOD1 aggregate seeds, prepared either from Tg mice or from a patient carrying the hSOD1^G127X^ mutation, transmitted spreading templated hSOD1 aggregation and premature motor neuron disease to hSOD1^G85R^ Tg model mice [9, 13]. Here we set out to examine whether seeds from postmortem ventral horns from patients carrying the most prevalent hSOD1 mutation, D90A, also would transmit aggregation and disease to hSOD1^G85R^ Tg mice. To this end, seeds were prepared from lamina IX of ventral horns from six patients homozygous for the hSOD1^D90A^ mutation (Table 1). One µl of the seeds was inoculated into lumbar ventral horns of hSOD1^G85R^ Tg mice. Two of the preparations were found to significantly shorten the survival of recipient mice compared with non-inoculated hSOD1^G85R^ mice; A2 and A1 (Fig. 1a, b, Table 3). In contrast, inoculation of nine different seeds from four different control individuals resulted in survivals close to those of non-inoculated mice (Fig. 1c, d; Table 4). Three different seed preparations from C57BL/6 control mice likewise failed to influence the lifespan of hSOD1^G85R^ Tg mice (Fig. 1 d, Supplementary Fig. S2).

**Table 3:** Average time from inoculation to death in recipient mice for ALS patient inoculates

	D90A patient inoculates						
	Non-inoculated (*n*=86)	A1 (*n*=10)	A2 (*n*=12)	A3 (*n*=9)	A4 (*n*=8)	A5 (*n*=10)	A6 (*n*=5)
Days after inoculation ± standard deviation	297 ± 40^#^	274 ± 36 *(*p* = 0.040)	233 ± 40 ***(*p* <0.001)	306 ± 46 n.s. *p* = 0.312)	308 ± 48 n.s. (*p* = 0.354)	282 ± 33 n.s. (*p* = 0.121)	288 ± 46 n.s. (*p* = 0.693)

Symbols denote significance of differences between patient seeds inoculated and non-inoculated mice: ns, *p* > 0.05; *, *p* ≤ 0.05; **, *p* ≤ 0.01; ***, *p* ≤ 0.001; ****, *p* ≤ 0.0001 (log-rank Mantel Haenszel test). #, The survival length of the non-inoculated mice was subtracted with 102 d, the mean age at which the mice were inoculated

**Table 4:** Average time from inoculation to death in recipient mice for control patient inoculates. The lifespan for the non-inoculated mice was subtracted with 102 days, the mean age at which the mice were inoculated. I, II, and III refer to protocols for seed preparation

Patient inoculate	Average time from inoculation to death (days) ± standard deviation
Non-inoculated − 102d (*n*=86)	297 ± 40
C1 I (*n*=8)	285 ± 31 (*p* = 0.209)
C1 II (*n*=18)	296 ± 33 (*p* = 0.588)
C1 III (*n*=5)	290 ± 45 (*p* = 0.732)
C2 II (*n*=4)	329 ± 29 (*p* = 0.340)
C2 III (*n*=5)	303 ± 39 (*p* = 0.967)
C3 II (*n*=8)	303 ± 71 (*p* = 0.202)
C3 III (*n*=7)	302 ± 21 (*p* = 0.762)
C4 II (*n*=11)	309 ± 41 (*p* = 0.499)
C4 III (*n*=9)	315 ± 45 (*p* = 0.217)

### Structure of aggregates and spread along the neuraxis

**Fig. 2:**
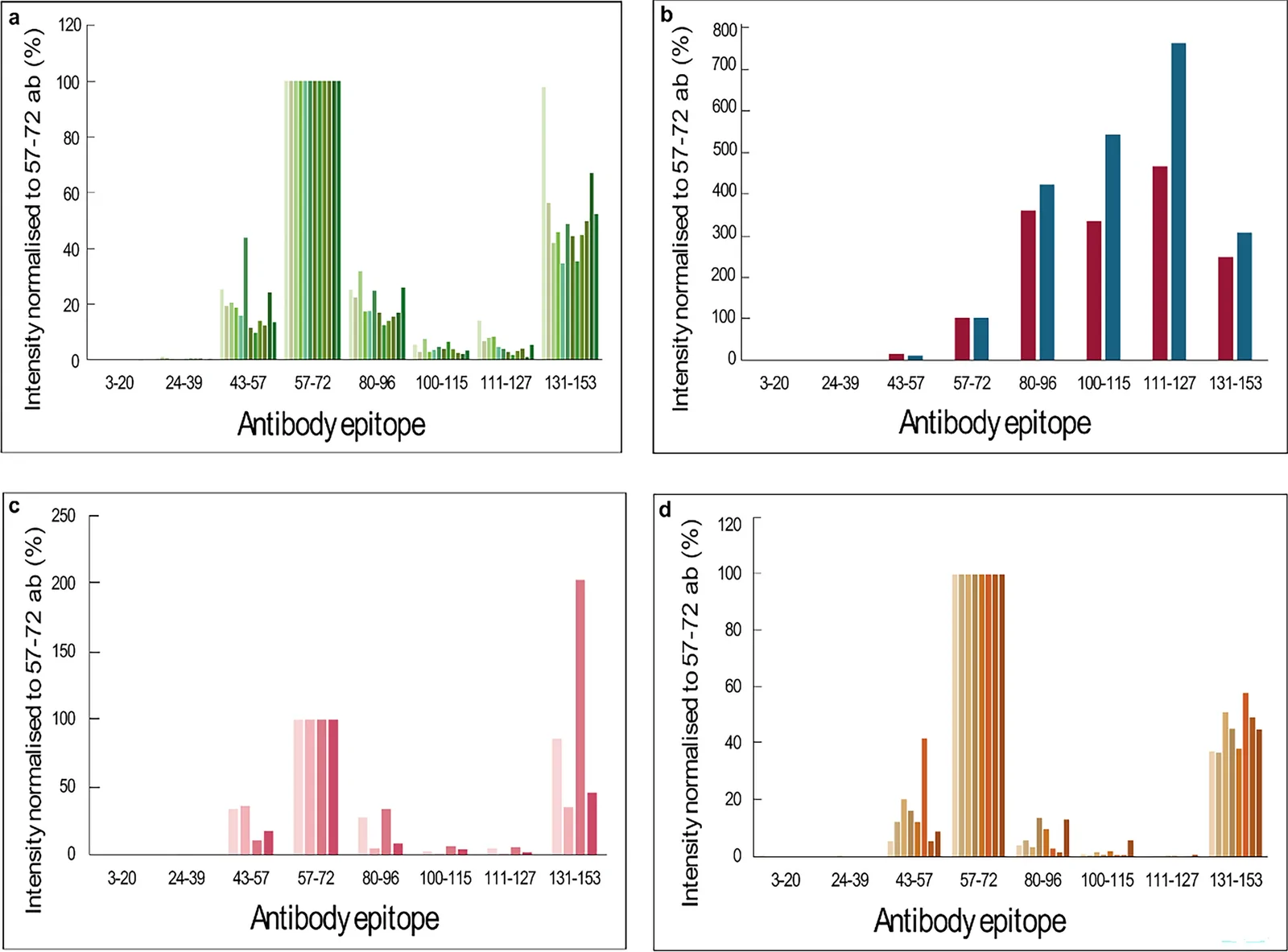
BEM patterns of spinal cords of terminally ill inoculated Tg mice. The data were normalized against the staining intensities of the cords against the aa 57–72 antibody.** a** Patterns of mouse spinal cords inoculated with the A2 seed. The data are presented in the order of survival with the most short-lived to the left. **b** Spinal cords of the two most short-lived mice inoculated with the A1 seed (A1:1 blue and A1:2 red). The specimens showed typical strain B patterns.** c** Patterns of spinal cords of the more long-lived mice inoculated with the A1 seed showing strain A patterns. **d** Patterns of the two most short-lived mice inoculated with the A3, A4, A5 and A6 seeds, all showing A patterns

**Fig. 3:**
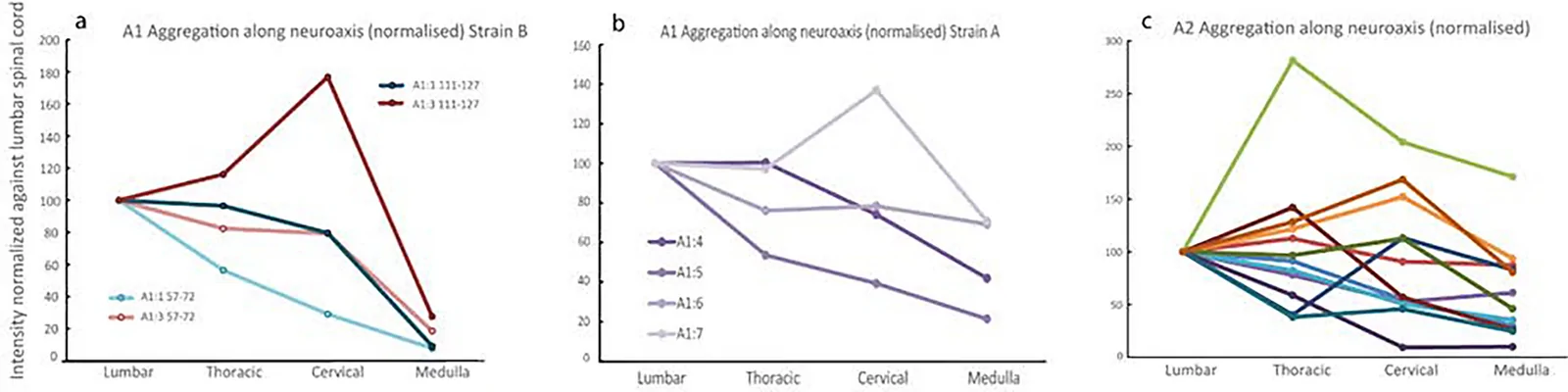
Distribution of aggregation along the neuraxis in terminally ill inoculated mice. The aggregate levels in the segments of the individual terminally ill mice were normalized against their levels in lumbar spinal cord. **a** Distribution of strain A (and B, aa 57–72 antibody) and strain B (aa 111–127 antibody) in the A1-inoculated mice showing the presence of strain B aggregates (A1:1 and A1:2). **b** Distribution of strain A aggregation analyzed with the aa 57–72 antibody in the A1:3, A1:4, A1:5 and A1:6 mice. **c** Distribution of strain A aggregation analyzed with the aa 57–72 antibody in the A2:1 to A2:12 mice

Spinal cord homogenates from terminal mice inoculated with human-derived seeds were analyzed with the 8-antibody BEM (Fig. 2). Those inoculated with the most effective seed, A2, showed all strain A patterns (Fig. 2a). The lifespan of mice inoculated with the A1 seed overlapped with those of non-inoculated and control-inoculated mice. Here the two most short-lived mice showed typical strain B patterns (A1:1 and A1:2) (Fig. 2b) whereas the remainder showed strain A patterns (Fig. 2c). One of these samples showed unusually high reactivity with the a 131–153 antibody, for which we have no explanation. The aggregations were found to be spread along the neuraxis in the terminally ill mice (Fig. 3a–c).

If any of the additional four seeds had transmitted any B aggregation, that would most likely appear in the most short-lived mice in the groups. We analyzed such mice, but only strain A patterns appeared (Fig. 2d). Admixture of even minimal amounts of strain B aggregates together with strain A aggregates would easily have been discerned [8]. Mice inoculated with one of the human control seeds (C1) were analyzed and likewise showed strain A patterns (Supplementary Fig. S3).

Lumbar inoculation of seeds that induce prion-like spread of hSOD1 aggregation typically cause hindleg symptom onsets [9, 13]. Non-inoculated hSOD1^G85R^ Tg mice, and mice inoculated with control preparations show onsets varying between hindleg, hindlegs and forelegs simultaneously, and forelegs and other anterior symptoms, likely reflecting random location of aggregation initiation along the neuraxis. In the A2 and A1 groups, lumbar onsets were seen in 11 of 12 and all 10 mice, respectively. In mice inoculated with seeds that did not cause significantly shortened survivals (A3, A4, A5, and A6), lumbar onsets were found in 21 of 32 (65.6%). In the combined onset data available from mice inoculated with the nine control seed preparations, lumbar onsets were seen in 39 of 62 individuals (62.9%). 

### Histopathology

**Fig. 4:**
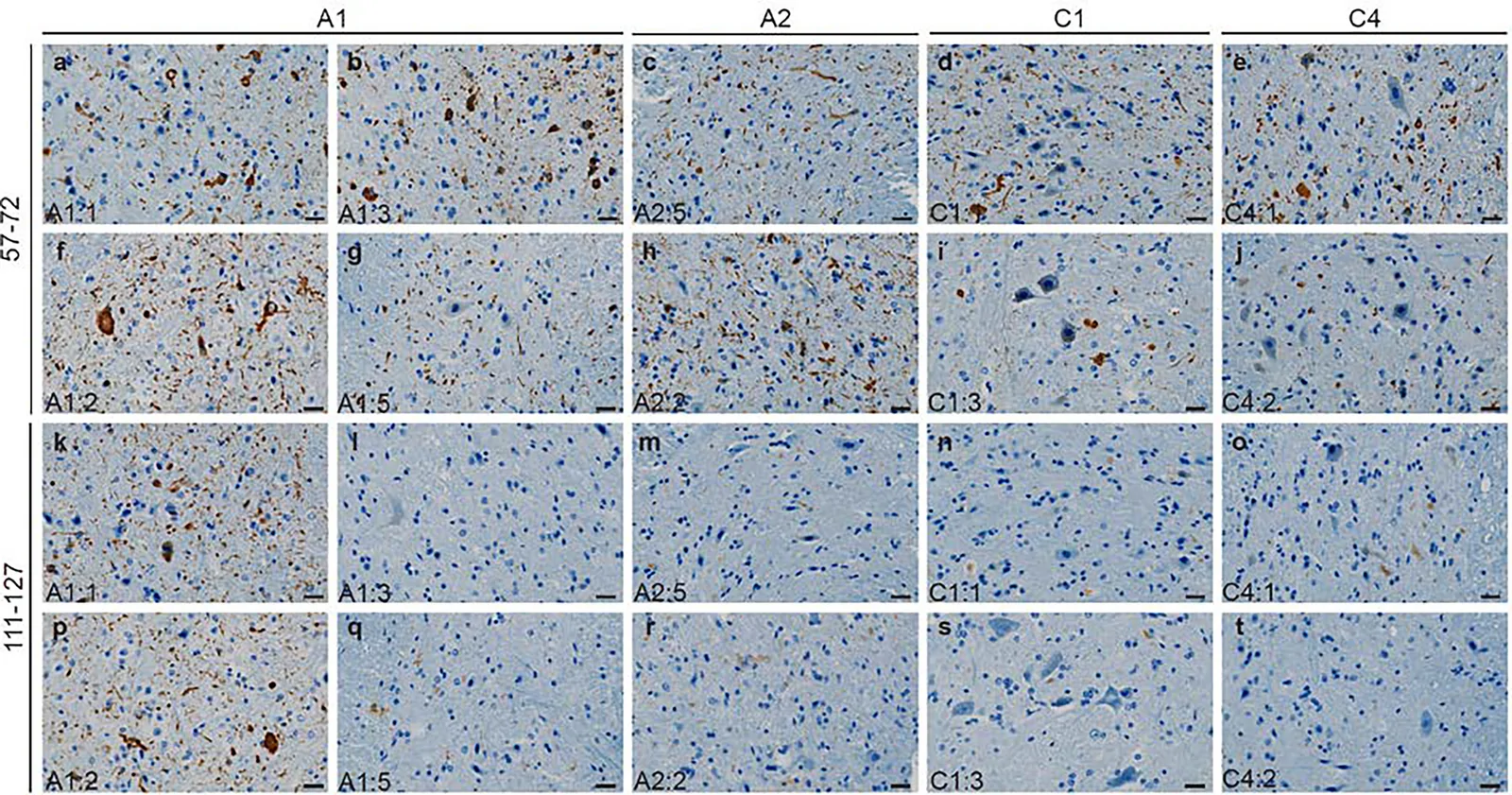
Immunolabeling of hSOD1 aggregates in mice inoculated with patient seeds. Spinal cord sections derived from mice inoculated with the A1, A2 or control patient seeds were immunolabeled with an antibody targeting hSOD1 aa 57–72 (**a**–**j)**, detecting both strain A and strain B, or an antibody targeting hSOD1 aa 111–127 (**k**–**t**), detecting strain B. Identities of analyzed mice, based on post-inoculation survival length, as previously described (see Methods), are shown in each image. All mice show aggregates reactive for the aa 57–72 antibody (**a**–**j**). However, only two mice, inoculated with seeds prepared from patient A1 (A1:1 and A1:2), show ample staining against aa 111–127 (**k**, **p**). Micrographs were obtained of the lumbar ventral horn of all mice. Scale bars represent 20 µm

To further examine the appearance of strain A and B aggregates, sections of spinal cord were stained with the aa 57–72 (stains both strains A and B) and aa 111–127 (stains only strain B) antibodies. In DAB-stained sections, specimens from both A2 and A1 mice show abundant aggregates using the aa 57–72 antibody (Fig. 4). In contrast, using the aa 111–127 antibody only two specimens from the A1-inoculated mouse (A1:1 and A1:2, not A1:3 and A1:5) displayed ample aggregate staining.

The specificity of the aa 111–127 antibody for staining B was validated in Supplementary Fig. S4. There was ample staining of aggregates in a terminally ill homozygous hSOD1^D90A^ mice, and in hSOD1^G85R^ mice inoculated with seed from a young terminal homozygous hSOD1^D90A^ Tg mouse. There was no staining of aggregates in a terminal hSOD1^G85R^ mouse nor in a terminal hSOD1^G85R^ mouse inoculated with a seed prepared from a terminal hSOD1^G85R^ mouse.

**Fig. 5:**
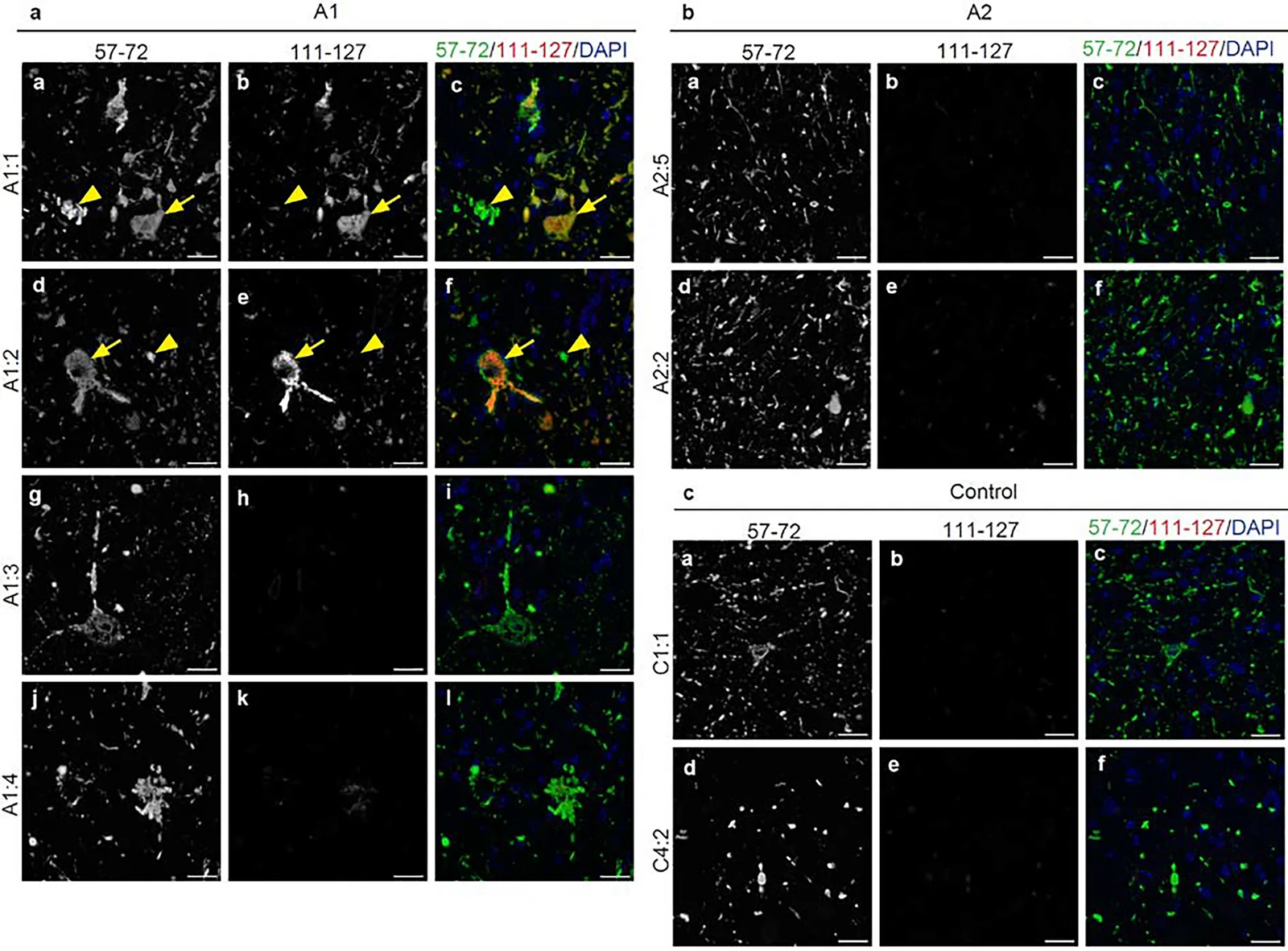
Immunolabeling of strain A and strain B hSOD1 aggregates in mice inoculated with patient seeds. Spinal cord sections derived from mice inoculated with the A1, A2 or control patient seeds were immunolabeled with antibodies targeting hSOD1 aa 57–72 (green) and hSOD1 aa 111–127 (red), and counterstained with DAPI (blue). **a** Two of the A1-inoculated mice (A1:1 and A1:2) display aggregates reactive with both antibodies (**a**–**c**, **d**–**f**, arrow), demonstrating strain B, and aggregates only reactive with the aa 57–72 antibody (**a**–**c**, **d**–**f**, arrowhead), demonstrating strain A. The other two mice inoculated with the same seed (A1:3 and A1:5) only show aggregates reactive for the aa 57–72 antibody (**g**–**i**, **j**–**l**). **b** The mice inoculated with seeds derived from patient A2 (A2:1 and A2:5) only show aggregates reactive with the aa 57–72 antibody (**a**–**c**, **d**–**f**), demonstrating strain A aggregates. **c** Control inoculated mice only show aggregates reactive for the aa 57–72 antibody (**a**–**c**, **d**–**f**), demonstrating strain A aggregates. Confocal images were obtained of the lumbar ventral horn of all mice. Scale bars represent 20 µm

For more detailed examinations, confocal fluorescence staining was used (Fig. 5). Specimens from the two A1-inoculated mice with strain B BEM patterns (A1:1 and A1:2) show the presence of both strain B and strain A aggregates in the tissue. In contrast specimens from two each of A1-inoculated mice with strain A BEM patterns (A1:3 and A1:5), A2-inoculated mice (A2:1 and A2:2), and control-inoculated mice show only the presence of strain A aggregates in the spinal cord.

## Discussion

Even though previous reports have demonstrated prion-like seeding effects in CNS preparations from patients with three different heterozygous SOD1 mutations [13, 30], little is understood regarding the primary ALS pathogenesis in humans carrying the more than 235 SOD1 mutations so far detected. Here we examined patients homozygous for the most prevalent mutation, D90A. Owing to the long disease course and dominating lower motor neuron lesion in such patients [3], the ventral horns become profoundly depleted of motor neurons, and seeds prepared from lamina IX are expected to contain little material from motor neurons [16] and could lead to low seeding activity. Indeed, the seeds contained only pg amounts of aggregated SOD1 per µl and some 50,000 times more of other ventral horn proteins (Table 1). Still, we found that seeds from two of the six patients induced premature ALS-like disease and shortened the lifespans of inoculated Tg mice. In contrast, none of the human control seed preparations affected the lifespan of hSOD1^G85R^ Tg mice, nor have various control mouse preparations done so (Fig. 1c, d; Supplementary Fig. S2; Table 4). The postmortem interval was significantly shorter in the control group (*p* = 0.019), which strengthens the validity of our findings. As seeding-competent material is sensitive to proteolytic degradation [21], its integrity is likely to be better preserved in samples with shorter postmortem intervals. The results indicate that lumbar spinal cord inoculation of seeds containing heterologous or homologous ventral horn tissue components lack effects on hSOD1 aggregation in Tg mice, unless prion-competent SOD1 aggregates are present.

We have previously reported that CNS tissue from old control subjects and homozygous D90A patients contains comparable amounts of detergent-insoluble SOD1 aggregates [19]. In the present study there was no obvious difference in total SOD1 aggregate amounts between the active A1 and A2 seeds and those without effect, A3-A6, nor any clear difference versus the control preparations. This suggests that the prion-active aggregates account for only subfractions of the total amount of detergent-insoluble SOD1 aggregates in the seed preparations.

The structures of prion-active aggregates are conceivably of great interest, e.g. for choice of epitopes in development of antibody-based therapies and other structure-dependent interventions. Moreover, the ALS phenotypes vary considerably between different *SOD1* mutations and the occurrence of different strains of SOD1 prions might explain part of this variability. Using BEM, we found that two different patterns, denoted A and B, can arise in the CNS in the mouse Tg models. The ventral horns of humans carrying hSOD1 mutations contain far less aggregates than the overexpressing Tg mice, and CNS from old humans contains large amounts of debris, including aggregated (wild type) hSOD1 [19]. This precludes meaningful analysis of mutant hSOD1 aggregate structures using BEM. However, hSOD1^G85R^ Tg mice seem to template the structure of inoculated aggregates, strain A results in formation of A aggregates and strain B in formation of B aggregates, even though B aggregates never develop spontaneously in this model [8, 9, 13].

The aggregates in all the A2-seed inoculated mice showed strain A BEM patterns, suggesting that the ventral horn of the patient contained strain A aggregates. The situation with A1 is more complex. We previously found that strain B aggregates propagate some 30% slower than strain A aggregates when inoculated into spinal cords of hSOD1^G85R^ Tg mice [9]. The B-transmitting A1 seed was seemingly less efficient than the A2 seed, and the survival curve of the inoculated mice overlapped extensively with those of non-inoculated mice which develop strain A aggregation [8, 9]. The reason why only the most short-lived mice contained B-aggregates is likely explained by the spontaneously arising strain A aggregation overwhelming any later seeded B aggregation. Notably, both strain A and strain B aggregates were found in the confocal study of the most short-lived mice. This could also be caused by the spontaneously arising strain A aggregation in the hSOD1^G85R^ Tg mice. However, homozygous hSOD1^D90A^ Tg mice typically contain both strain A and strain B aggregates [8], and our present results cannot exclude the possibility that the ventral horn of the A1 patient contained both these strains.

Seeds prepared from both hSOD1 Tg mice and human SOD1-ALS patients have been found to cause differences in rates of aggregation when inoculated into Tg mice. In a recent study, seeds from ALS patients with mutations associated with short survival also caused short lifespans in the Tg mice and vice versa [30]. In homozygous hSOD1^D90A^ Tg mice strain B dominates in mice with short lifespan and strain A in mice with longer lifespans [8]. The current B-transmitting seed was prepared from the D90A patient with the earliest onset and shortest symptomatic disease duration (A1), whereas the strain A transmitting seed was prepared from a patient with late onset and long disease duration. This sample of two patients is, however, too limited to allow any conclusions regarding disease phenotypes associated with the A and B SOD1 prion strains in D90A patients.

In conclusion, we find that seeds prepared from ventral horns from patients homozygous for the hSOD1^D90A^ mutation transmit premature motor neuron disease and spreading strain A or B aggregation in hSOD1^G85R^ Tg mice. While hSOD1^D90A^ shows wild-type-like molecular stability and enzymatic activity and is found in high concentration in the CNS [2, 19], the truncated hSOD1^G127X^ protein is inactive, disordered and present in only minute amounts in the CNS [17, 18]. Homozygous hSOD1^D90A^ patients show a uniform phenotype with slowly ascending progression following onset in a leg and live typically more than a decade [2, 3]. Human SOD1^G127X^ patients show variation in sites of onset and progression and live 2–4 years [4]. The fact that seeds from patients carrying hSOD1 mutations with so divergent characteristics transmit motor neuron disease and hSOD1 aggregation lends further support to the hypothesis that prion-like spread of hSOD1 aggregation generally could be the primary pathogenic mechanism in hSOD1-induced ALS.

## Supplementary Information

Below is the link to the electronic supplementary material.Supplementary file1 (DOCX 6412 KB)

## Data Availability

Reasonable data sharing requests are made in writing through the corresponding author (e-mail) and require a formal data sharing agreement if of scientific interest and legally and ethically possible. Data sharing agreements must include details on how the data will be stored, who will have access to the data and intended use of the data, and agreements as to the allocation of intellectual property.
