# Supplementary material for: Seeds from ALS patients homozygous for the SOD1 D90A mutation transmit two types of SOD1 aggregation and motor neuron disease
Source: Acta Neuropathol. 2026 Sep 1;152(1):27. doi: 10.1007/s00401-026-03078-3 (PMC13534153; doi:10.1007/s00401-026-03078-3)
Supplement: Supplementary file 1 [file 401_2026_3078_MOESM1_ESM.docx]

**Supplementary data**

**Seeds from ALS patients homozygous for the SOD1 D90A mutation transmit two types of SOD1 aggregation and motor neuron disease**

Caitlin Henne^1^, Isabelle Sigfridsson^1^, Thomas Brännström^1^, Karin M. E. Forsberg^2^, Stefan L. Marklund^1^, Per Zetterström^1^, Peter M. Andersen^2^

^1^Department of Medical Biosciences, Umeå University, 9901 87 Umeå, Sweden

^2^Department of Clinical Sciences, Neuroscience, Umeå University, 901 87 Umeå, Sweden

Correspondence to: Stefan L. Marklund

E-mail: [Stefan.marklund@umu.se](mailto:Stefan.marklund@umu.se)

Telephone: +46 705687703

**a**

**b**

**Supplementary Fig. S1** Examples of blots in the BEM analysis. The dilutions made of the primary 1+25 spinal cord homogenates are shown in the figure. The standard was prepared from spinal cords of end-stage hSOD1^G93A^ Tg mice handled in the same way (c.f. Materials and methods). 3-20 etcetera refer to the anti-hSOD1 peptides used for preparing the antibodies used in the blots. **a** BEM blot of homogenate of lumbar spinal cord from the A1:1 mouse showing strain B pattern. **b** BEM blot of homogenate of lumbar spinal cord from the A1:6 mouse showing strain A pattern.

**Supplementary Fig. S2** No significant effect on survival of hSOD1^G85R^ mice inoculated with a seed prepared from C57Bl6 control mice. The seed was prepared according to a protocol similar to II, except that the preparation was sedimented onto a 75.5% iohexol cushion under the 13% iohexol. The survival length of the non-inoculated mice (n = 86) was subtracted with 102 d, the mean age at which the mice were inoculated.

**Supplementary Fig. S3** BEM profiles of mice inoculated with the C1 control seed showing strain A patterns.

**Supplementary Fig. S4** Immunohistochemical validation of the aa 111-127 antibody specificity. Negative controls (expected to contain A-aggregates), consisting of tissue derived from a terminal hSOD1^G85R^ mouse (**a**) and a terminal hSOD1^G85R^ mouse inoculated with a seed prepared from a terminal hSOD1^G85R^ mouse (**b**), show no or sparse staining with the aa 111-127 antibody. Positive controls (expected to contain B-aggregates), consisting of tissue derived from a terminal homozygous hSOD1^D90A^ mouse (**c**) and a hSOD1^G85R^ mouse inoculated with a seed prepared from a young terminal hSOD1^D90A^ mouse (**d**), show ample staining against the 111-127 epitope. Images are obtained in the ventral horn of the spinal cord. Scale bars represent 20 µm.
